# Risk factors of mild behavioral impairment: a systematic review

**DOI:** 10.3389/fpsyg.2025.1586418

**Published:** 2025-06-27

**Authors:** Song Lin Tang, Ponnusamy Subramaniam, Ching Sin Siau, Agnes Shu Sze Chong, Fang Liu

**Affiliations:** ^1^Department of Geriatrics, Zhoushan Hospital, Wenzhou Medical University, Zhoushan, China; ^2^Center for Healthy Ageing and Wellness, Faculty of Health Sciences, Universiti Kebangsaan Malaysia, Kuala Lumpur, Malaysia; ^3^Center for Community Health Studies, Faculty of Health Sciences, Universiti Kebangsaan Malaysia, Kuala Lumpur, Malaysia

**Keywords:** mild behavioral impairment, risk factors, systematic review, cognitive decline, Alzheimer’s disease

## Abstract

**Introduction:**

Mild Behavioral Impairment (MBI) represents a predementia syndrome marked by neuropsychiatric symptoms that may precede detectable cognitive decline. Identifying factors associated with MBI is critical for developing targeted prevention strategies in neurodegenerative disorders.

**Methods:**

This systematic review adhered to PRISMA 2020 guidelines, searching PubMed, Scopus, Web of Science, ScienceDirect, and Embase through May 2024. Forty-one human studies meeting predefined inclusion criteria were selected through dual independent screening.

**Results:**

Five key domains emerged: (1) Genetic susceptibility (APOE ε4 allele showing strongest association), (2) Motor system pathology (particularly Parkinsonian features), (3) Multisensory deficits (auditory impairment demonstrating bidirectional relationships), (4) Metabolic dysregulation (diabetes mellitus and frailty phenotypes), and (5) Neuroanatomical correlates (frontolimbic atrophy patterns on MRI). The interaction between genetic predisposition and environmental/lifestyle factors appears central to MBI pathogenesis.

**Conclusion:**

MBI manifests as a multidimensional interface between molecular mechanisms and clinical phenomenology. Our synthesis supports the implementation of transdiagnostic screening protocols integrating behavioral biomarkers with conventional cognitive assessments. Future research should prioritize longitudinal designs to establish causal pathways and intervention thresholds.

## 1 Introduction

Mild Behavioral Impairment (MBI) is a recently defined concept that identifies behavioral and psychological changes suggestive of underlying neurodegenerative processes, often appearing before the diagnosis of major neurocognitive disorders like Alzheimer’s disease (Martin and Velayudhan, 2020). Proposed in 2003 (Taragano and Allegri, 2003) and refined in 2016 (Ismail et al., 2016), MBI can serve as an early indicator of Alzheimer’s disease (AD).

MBI is a prodromal syndrome characterized by late-life neuropsychiatric symptoms (NPS) that persist for at least 6 months, reflect a change from the individual’s baseline behavior, and are not explained by traditional psychiatric diagnoses (Ismail et al., 2017). Neuropsychiatric symptoms, such as mood and behavioral changes, are early manifestations in predementia stages and are associated with an increased risk of cognitive decline and clinical conversion from normal cognition to MCI and from MCI to dementia. Historically, these symptoms have been recognized as significant indicators of impending cognitive decline, highlighting the need for early detection and intervention. It is important to differentiate MBI from MCI, which is characterized by noticeable cognitive decline that does not significantly interfere with daily life. While MCI can progress to dementia, MBI focuses on behavioral changes that may precede cognitive decline. Understanding these distinctions is crucial for developing effective early intervention strategies.

The ISTAART diagnostic criteria provide a framework for identifying MBI and its associated neuropsychiatric symptoms, emphasizing the importance of early detection and intervention (Ismail et al., 2016). As the global population ages, the importance of MBI grows, especially given the potential for early intervention and disease modification (Pan et al., 2021). Identifying MBI at its nascent stage could be pivotal in preventing the progression to dementia and in recognizing individuals who develop neuropsychiatric symptoms early, ultimately facilitating the development of more targeted and effective preventive therapies (Jin et al., 2023).

Early detection of MBI is crucial for preventing or delaying the progression to dementia (Jiang et al., 2022). It enables timely implementation of interventions such as cognitive training, lifestyle modifications, which can slow down the neurodegenerative process, improve cognitive function, and enhance the quality of life of affected individuals. A growing body of research has examined various factors associated with MBI, such as genetic predispositions, biomarkers, and socio-demographic influences. Recent studies, such as those by Matsuoka et al. (2023), have explored neuroimaging findings related to MBI and suggested its association with pathological changes leading to dementia. Specifically, MRI studies have shown structural changes in brain regions like the prefrontal cortex (Ghahremani et al., 2023a) and hippocampus (Johansson et al., 2021) in MBI participants. These regions are crucial for cognitive and emotional regulation, and their atrophy or altered connectivity may contribute to the emergence of neuropsychiatric symptoms even before cognitive decline becomes apparent. Although some factors have been investigated, a comprehensive understanding of various risk factors such as demographic variables, genetic factors, comorbid conditions, cognitive status, and psychosocial influences is still lacking.

Previous reviews, such as the mini review by Creese and Ismail (2022), have summarized the emerging clinical and biomarker evidence related to MBI. Their work focused on the measurement and clinical correlates of MBI as a marker of preclinical Alzheimer’s disease, but it did not delve into demographic, comorbid, or psychosocial risk factors in depth. Our current review builds on this by providing a more in-depth analysis of a wider range of risk factors associated with MBI. By addressing these factors, we hope to improve outcomes for individuals at risk.

## 2 Methods

This review was conducted in accordance with the PRISMA 2020 statement (Page et al., 2021), and the protocol was registered with PROSPERO (https://www.crd.york.ac.uk/PROSPERO/view/CRD42024534858, CRD42024534858).

### 2.1 Search strategy

A systematic literature search was performed using PubMed, Scopus, Web of Science, ScienceDirect, and Embase for studies published from inception to 10 May 2024. Google Scholar was also utilized to identify relevant studies that were not captured in these databases. The search terms included (“Cognitive Dysfunction,” “Cognitive Impairments,” “Cognitive Disorder,” “Mild Cognitive Impairment,” “Cognitive Decline,” “Mental Deterioration,” “Neurocognitive Disorders,” “Neuropsychiatric Symptoms”) and (“Mild Behavioral Impairment” OR “Mild Behavioral Impairment” OR “Mild Behavior Impairment”). The above-mentioned terms are only examples for retrieval. The complete search strategies for each database (including combinations of Boolean operators and MeSH terms) are detailed in Supplementary material S1. The analysis was restricted to articles published in English. Additional studies were identified from the reference lists of the included studies and relevant reviews.

### 2.2 Screening process

The screening process was meticulously conducted in two distinct phases by two independent reviewers, SLT and SCS, with oversight from the review team (ACSS and PS). Initially, both reviewers independently screened titles and abstracts to identify potential candidates for inclusion. Following this initial phase, the full texts of the shortlisted articles were obtained for a more comprehensive assessment. Any discrepancies between the reviewers’ decisions were resolved through discussion or, if necessary, by consulting a third review author, PS.

Eligible studies were those that met the following inclusion criteria: (1) original observational research papers involving human subjects, with a prospective, retrospective, or cross-sectional design; (2) studies providing information to identify risk factors for Mild Behavioral Impairment (MBI) among human subjects; and (3) studies published in English. Exclusion criteria were applied to studies that: (1) did not involve risk factors of MBI or MBI diagnosis; (2) included animal experiments, qualitative studies, case reports, reviews, abstracts from conferences, posters, theses, protocols, editorials, letters, and book chapters; and (3) only considered MBI as a precursor stage in the development of other specific diseases (such as Parkinson’s disease, Alzheimer’s disease, etc.) and do not conduct independent analyses of MBI’s own risk factors.

### 2.3 Data extraction

Data were extracted and synthesized independently by SLT and SCS using a Microsoft Excel spreadsheet, following a predetermined data extraction template. All information was in the form of open - text responses. Additionally, before the start of the study, we did not screen for specific categories of risk factors. The following study characteristics were extracted: (1) name of the first author; (2) publication year; (3) country; (4) study design; (5) study setting; (6) participants’ information related to the study (e.g., population types involved, including Parkinson’s disease patients, Alzheimer’s disease patients, cognitively normal older adults); (7) number and type of subjects; (8) mean age or age range; (9) proportion of female subjects; and (10) research data with the quantitative indicators of factors related to MBI, such as odds ratio (OR value), p - value, etc.; (11) assessment tool for MBI; (12) factors associated with MBI; and (13) main outcome. Any disputes regarding the extracted data were resolved through consensus or consultation with the principal investigator, PS. The Rayyan website and EndNote reference management software were utilized to efficiently manage and organize the articles included in the review.

### 2.4 Quality assessment

Critical assessment instruments developed by the Joanna Briggs Institute (JBI) for cross-sectional and cohort research (Moola et al., 2020) were employed to assess the quality of the selected studies (Supplementary material S2). Key domains included study design validity (e.g., clear inclusion criteria, baseline comparability), measurement quality (valid/reliable exposure/outcome assessment), confounding management (identification and statistical adjustment), and follow-up adequacy (for cohort studies). One reviewer (SLT) applied these checklists, rating each item as “Yes,” “No,” “Unclear,” or “Not Applicable,” and the results were reviewed by the other researchers (PS, ASZC, and CSS).

## 3 Results

**Figure 1:**
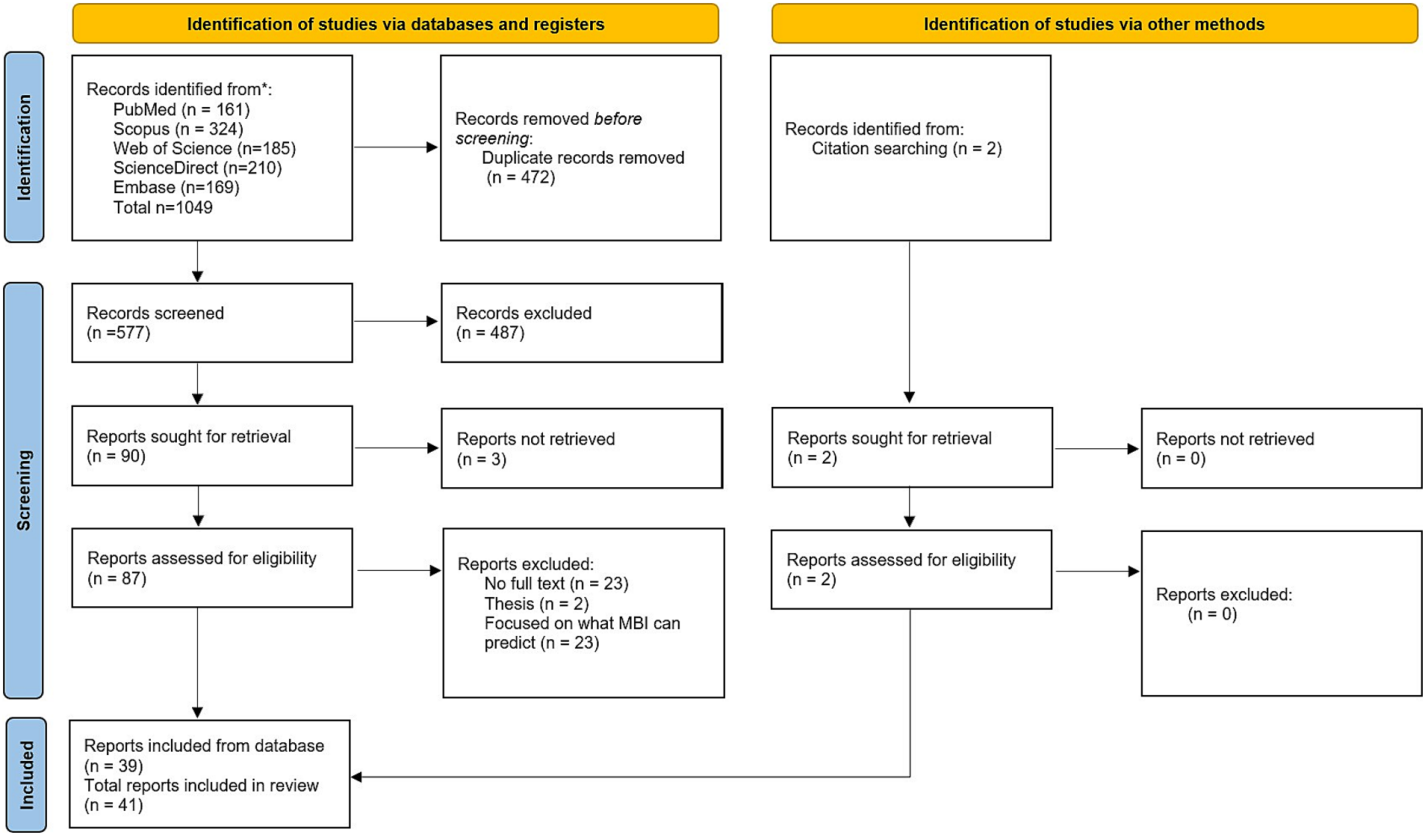
PRISMA flow diagram. This diagram illustrates the screening process of records and reports. Reports included from database screening are 39, and through citation searching, an additional 2 reports are identified, leading to a total of 41 reports included in the review. MBI refers to Mild Behavioral Impairment.

The initial database search identified 1,049 citations. After removing duplicate records, 577 titles and abstracts were screened for eligibility, resulting in 90 eligible citations for full-text retrieval. Two additional citations were identified through hand searching the reference lists of the included articles. In total, 41 studies met the eligibility criteria for this systematic review. Figure 1 illustrates the selection process using a PRISMA flowchart.

### 3.1 Study characteristics

Table 1 provides basic details on the 41 studies that met the established criteria. The participants’ ages ranged from 44 to 100 years. Although the ISTAART criteria for MBI define symptom onset at age 50 or older, risk factors may emerge earlier. This study aims to identify these risk factors, which is why participants as young as 44 years were included. Most of these studies focused on normal cognition (NC), subjective cognitive decline (SCD), or mild cognitive impairment (MCI), while some neuroimaging studies concentrated on Parkinson’s disease (PD) or Alzheimer’s disease (AD). Eight studies (Andrews et al., 2018; Mortby et al., 2018; Gosselin et al., 2019; Gosselin et al., 2022; Creese et al., 2023; Leow et al., 2024; Richey et al., 2024; Wolfova et al., 2022) were conducted in community settings, five studies (Soo et al., 2021; Cassidy et al., 2022; Mudalige et al., 2023; Ismail et al., 2023; Tsai et al., 2023) included both community and clinical settings, and the remaining 28 studies were conducted in clinical settings.

**Table 1:** An overview of studies.

Authors and publication year	Country	Study setting	Study design	Participants(N)	Age, range or mean +/− SD	Gender (Percent Female)
Andrews et al. (2018)	Australia	Community	Cross-sectional data from cohort study	1,226 CN = 763 CN-AR = 352 MCI = 111	72–79	39.87%
Mortby et al. (2018)	Australia	Community	Cross-sectional	1,377 CN = 847 CN-AR = 397 MCI = 133	72–79	48%
Baschi et al. (2019)	Italy	Clinic	Cross-sectional	429 PD	68.2 ± 9.4	40.10%
Gosselin et al. (2019)	Canada	Community	Prospective study	35 HC	60–93	49%
Yoon et al. (2019)	Canada	Clinic	Cross-sectional	60 PD, 29 healthy controls	58–81	37.08%
Fan et al. (2020)	China	Clinic	Cross-sectional	137	60–90	68.60%
Lang et al. (2020)	Canada	Clinic	Cross-sectional	102 74 on-demented PD,28 HC	71.8 ± 6.4	39.20%
Lussier et al. (2020)	Canada	Clinic	Cross-sectional	96 CN older adults	57–85	60.40%
Rao et al. (2020a)	India	Clinic	Cross-sectional	124 older adults	69.21 ± 6.64	28.23%
Rao et al. (2020b)	India	Clinic	Cross-sectional	124 older adults	69.21 ± 6.64	28.23%
Yoo et al. (2020)	South Korea	Clinic	Cross-sectional	275 PD	PD-MBI-66.26 ± 8.88 PD-MBI + 68.67 ± 8.31	49.80%
Ramezani et al. (2021)	Canada	Clinic	Cross-sectional	146 PD patients at H&Y II–III	47–86	36% female in Val carriers, 48% in Met carriers.
Gill et al. (2021)	Canada	Clinic	Cross-sectional data from cohort study	203 NC = 70, MCI = 95, AD = 38	70.30 ± 7.67	45.32%
Johansson et al. (2021)	Sweden	Clinic	Cross-sectional	50 amyloid-β-positive cognitively unimpaired subjects	44–88	50%
Matsuoka et al. (2021)	Japan	Clinic	Cross-sectional	43 (30 CN, 13 with aMCI)	76.9 ± 5.7	53.50%
Miao et al. (2021)	France	Clinic	Cross-sectional data from cohort study	768 MCI	72.75 ± 8	57.50%
Shu et al. (2021a)	China	Clinic	Cross-sectional	70 CN (32 with MBI, 38 HC)	NC with MBI 67.3 ± 6.6 NC without MBI 66.3 ± 7.3	54.29%
Shu et al. (2021b)	China	Clinic	Cross-sectional	34 CN (16 MBI patients and 18 HC)	NC with MBI 67.31 ± 6.69 NC without MBI 66.67 ± 7.18	52.94%
Soo et al. (2021)	Singapore	Community&Clinic	Cross-sectional	172 (79 CN and 93 MCI)	CN 63.86 ± 7.79 MCI 69.08 ± 7.75	43.60%
Yoon et al. (2021)	Canada	Clinic	Cross-sectional	85 older adults. 59 PD and 26 HC	58–82	42.35%
Bray et al. (2022)	United States	Clinic	Cohort study	946, with 124 having a history of TBI	TBI 76.45 ± 8.91 No TBI 77.52 ± 9.15	61.31%
Cassidy et al. (2022)	Canada	Community&Clinic	Cross-sectional	190 (118 CN, 44 MCI, 28 AD)	CN 72.3 ± 5.7 MCI 73.2 ± 5.4 AD 67.4 ± 8.9	36.32%
Matuskova et al. (2021)	Czechia	Clinic	Cross-sectional	116 with SCD	69.56 ± 8.18	49%
Gosselin et al. (2022)	Canada	Community	Cross-sectional	219	50–87	49%
Miao et al. (2022)	Canada	Clinic	Cross-sectional data from cohort study	139 86 NC and 53 MCI	55–90	51.80%
Stella et al. (2022)	Brazil	Clinic	Cross-sectional	80 older adults. 65 MCI and 15 CN	aMCI 74.5 ± 7.2 mdMCI 74.3 ± 6.8 NC 72.3 ± 8.5	72.50%
Guan et al. (2022)	Canada	Clinic	Cross-sectional	219	50–90	49%
Wolfova et al. (2022)	United Kingdom	Community	Cohort database	8,181	mean age 63	73%
Yang et al. (2022)	China	Clinic	Cross-sectional	60 NC	69.45 ± 7.38	51.67%
Creese et al. (2023)	United Kingdom	Community	Cohort study	2,750 no MBI = 2,499, MBI = 251	no MBI 64 ± 6.8 MBI 63 ± 7.0	73.96%
Ghahremani et al. (2023a)	Canada	Clinic	Cross-sectional data from cohort study	95 with 32 MBI + and 63 MBI– participants	71.7 ± 7.4	54.70%
Gosselin et al. (2023)	Canada	Clinic	Cohort database	7,080	50–100	61.70%
Ghahremani et al. (2023b)	Canada	Clinic	Cohort database	571	MBI 72.1 ± 7.25 NPSnoMBI 72.2 ± 7.14 No NPS 72.2 ± 7.03	46.80%
Mudalige et al. (2023)	United States	Community&Clinic	Cohort database	28,081	50–104	59.00%
Ismail et al. (2023)	United States and Canada	Community&Clinic	Cohort study	510 MCI, with 352 from ADNI and 158 from MEMENTO	ADNI 71.68 ± 7.4 MEMENTO 68.98 ± 8.18	44.30%
Tsai et al. (2023)	China	Community&Clinic	Cross-sectional	242 (129 aMCI, 113 CN)	71.6 ± 7.6	58.70%
Monchi et al. (2024)	Canada	Clinic	Cross-sectional	127 older adults 91 PD s and 36 HC	58–88	40.16%
Leow et al. (2024)	Singapore	Community	Cross-sectional data from cohort study	607	61.99 ± 10.19	57.60%
Matsuoka et al. (2024)	Japan	Clinic	Cross-sectional	80 participants (5 CN and 75 MCI)	78.5 ± 6.3	63.75%
Matuskova et al. (2024)	Czechia	Clinic	Cross-sectional	112 (62 aMCI-AD, 50 CN)	CN 67.30 ± 6.50 aMCI-AD 72.34 ± 4.96	59.82%
Richey et al. (2024)	United States	Community	Prospective cohort study	2,534	76	58.90%

*AD, Alzheimer’s disease; ADNI, Alzheimer’s Disease Neuroimaging Initiative; aMCI, Amnestic mild cognitive impairment; CN, Cognitively normal; CN-AR, Cognitively normal at risk; HC, Healthy controls; H&Y, Hoehn and Yahr stages; mdMCI, multiple-domain amnestic mild cognitive impairment; MEMENTO, French MEMENTO cohort study; MCI, Mild cognitive impairment; NC, Normal cognition; NPS, Neuropsychiatric symptoms; PD, Parkinson’s Disease; SCD, Subjective cognitive decline; TBI, Traumatic brain injury.

### 3.2 Quality assessment of included studies

Quality assessment of included studies showed that 28 were cross sectional studies and 13 were cohort studies. All studies met the basic methodological checks of the JBI tool (see Supplementary material S2), which included criteria such as clear inclusion/exclusion criteria, valid outcome measures, and appropriate statistical methods. While some studies had limitations (e.g., Mortby et al. (2018): item 5 ‘No’ for confounding adjustment; Gosselin et al. (2019): items 8 ‘No’ for follow-up adequacy), they were included as they met minimum validity thresholds for observational research.

### 3.3 Assessment methods

Table 2 outlines the methods used to assess MBI, including the MBI-Checklist (MBI-C) (Ismail et al., 2017a), the MBI criteria established by the International Society to Advance Alzheimer’s Research and Treatment - Alzheimer’s Association (ISTAART-AA) (Ismail et al., 2016), and The Neuropsychiatric Inventory (NPI) (Cummings et al., 1994). The NPI-Questionnaire (NPI-Q) (Kaufer et al., 2000) was also employed, utilizing a published algorithm (Mortby et al., 2018; Sheikh et al., 2018) to transform the NPI ratings into an MBI score.

**Table 2:** Assessment tool, associated factors and main findings of included studies.

Authors	Assessment tools of MBI	Associated factors	Main findings
Andrews et al. (2018)	ISTAART-AA MBI diagnostic criteria (Taragano and Allegri, 2003), NPI (Ismail et al., 2016)	Genetic risk (APOE ε4)	Affective dysregulation associated with genetic risk; APOE ε4 OR = 1.65 (1.2–2.25), *p* < 0.01.
Mortby et al. (2018)	ISTAART-AA diagnostic criteria, NPI	Gender, cognitive function	Higher prevalence of MBI in males; symptoms like reduced motivation were pronounced.
Baschi et al. (2019)	NPI	Motor function	Found associations between motor function and MBI symptoms in Parkinson’s Disease.
Gosselin et al. (2019)	MBI-C (Ismail et al., 2017a)	Comorbidities	MBI associated with comorbidity patterns; specific symptom prevalence noted.
Yoon et al. (2019)	MBI-C	Cognitive impairment	MBI + group had lower cognitive scores compared to healthy controls.
Fan et al. (2020)	MBI-C	Frailty	MBI + status linked to increased frailty risk (OR = 3.09, 95% CI = 1.29–9.41; *p* = 0.047); significant link to higher frailty risk without dementia.
Lang et al. (2020)	MBI-C	Education, UPDRS-III scores, connectivity	PD-MBI group had lower education levels [H(2,99) = 6.99, *p* = 0.03]; higher UPDRS-III scores; reduced functional connectivity between striatum and DMN [*F*(1,40.8) = 7.66, *p* = 0.0085].
Lussier et al. (2020)	MBI-C	SUVR of [18F] AZD4694	Significant correlation between MBI-C scores and SUVRs; global SUVR (R = 0.27, *p* < 0.0074), striatal SUVR (R = 0.3, *p* < 0.0028).
Rao et al. (2020a)	NPI-Q (Kaufer et al., 2000), ISTAART-MBI criteria	MCI type, marital status, urinary incontinence	Significant differences in MCI type, marital status, urinary incontinence, depression, multimorbidity, and diabetes between MBI and non-MBI groups (*p* < 0.05).
Rao et al. (2020b)	MBI-C, NPI-Q	Vitamin D, triglycerides	Significant correlation between low Vitamin D (*p* = 0.005) and high triglycerides (*p* = 0.044) with MBI.
Yoo et al. (2020)	ISTAART-AA MBI diagnostic criteria, NPI	UPDRS motor score, DAT availability	PD-MBI + group had higher UPDRS motor scores (20.27 ± 7.74 vs. 23.20 ± 9.37, *p* = 0.007); lower DAT availability in anterior caudate and putamen associated with MBI.
Ramezani et al. (2021)	MBI-C	BDNF Met allele	BDNF Met allele carriers had higher likelihood of being MBI positive (OR = 4.38, *p* = 0.002); associated with larger MBI load in PD.
Gill et al. (2021)	NPI-Q	Impulse dyscontrol symptoms	MBI impulse dyscontrol linked to reduced FA in fornix and superior frontal-occipital fasciculus; established atrophy patterns of AD.
Johansson et al. (2021)	MBI-C	Tau-PET activity, CSF P-tau181	Positive association between tau pathology and elevated MBI-C scores; MBI may indicate tau-related pathology in AD.
Matsuoka et al. (2021)	MBI-C	Functional connectivity	MBI-C negatively correlated with connectivity between left posterior parietal cortex and right middle frontal gyrus (*p* = 0.015).
Miao et al. (2021)	NPI-Q	White matter hyperintensity (WMH)	MBI + status showed 9.4% higher WMH volume compared to MBI- status (*p* = 0.01).
Shu et al. (2021a)	MBI-C	Network topology	Aberrant topological features in structural covariance networks; reduced local efficiency and clustering factors in MBI.
Shu et al. (2021b)	MBI-C	Education duration, brain volume	MBI group had shorter education duration and higher MBI-C scores; distinct brain shrinkage patterns linked to MBI.
Soo et al. (2021)	MBI-C	Diabetes mellitus (DM)	Higher occurrence of MBI in MCI individuals with DM (28.1% vs. 10.4%, *p* = 0.025); DM linked to increased MBI severity.
Yoon et al. (2021)	MBI-C	Activation in planning tasks	PD-MBI group showed reduced activation in planning tasks; significant correlation between hippocampus activation and MBI-C scores (*p* = 0.048).
Bray et al. (2022)	NPI-Q	TBI severity, LOC duration	Greater severity of TBI with LOC > 5 min linked to MBI before dementia onset (ORadj. = 4.034, *p* = 0.024); TBI severity linked to abnormal perception/thought content (HRadj. = 3.703, *p* = 0.005).
Cassidy et al. (2022)	MBI-C	Tau load, amyloid-β burden, cortical GMV	Larger mid-caudal LC signal predicted MBI severity (*p* = 0.019), especially impulse dyscontrol (*p* < 0.01) in tau-positive patients.
Matuskova et al. (2021)	MBI-C	Medial temporal lobe atrophy	Entorhinal cortex linked to MBI-C total score (*p* < 0.001); HV related to lower motivation (*p* = 0.008) and impulse dyscontrol (*p* = 0.011).
Gosselin et al. (2022)	NPI-Q	HHIE-S score	HHIE-S score significantly associated with higher global MBI symptoms, particularly in apathy (OR = 1.09, *p* = 0.002) and affective dysregulation (OR = 1.08, *p* < 0.001).
Miao et al. (2022)	NPI	Plasma A*β*42/Aβ40 levels	Reduced plasma Aβ42/Aβ40 correlated with elevated MBI (*p* = 0.04); age significant covariate (*p* = 0.003).
Stella et al. (2022)	MBI-C	Comorbidities, APOE ε4 allele	Increased comorbidities linked to higher MBI-C scores; APOE ε4 allele positively correlated with MBI-C scores, but not significant.
Guan et al. (2022)	NPI-Q	Frailty Index (FI)	Higher FI scores linked to MBI symptoms (OR = 2.73, *p* = 0.001); associations were sex-dependent, with males reporting higher severity.
Wolfova et al. (2022)	MBI-C	Gender	Males showed higher MBI levels (*p* < 0.05) and stronger associations with cognitive decline across MBI domains.
Yang et al. (2022)	MBI-C	White matter hyperintensities (WMH)	HWMH group had higher MBI-C scores (*p* < 0.05); WMH associated with atrophy of GMV and cortex, mediating MBI-C scores.
Creese et al. (2023)	MBI-C	APOE ε4 allele	MBI-psychosis linked to higher risk of cognitive impairment (HR = 3.6, *p* < 0.0001); APOE ε4 modifies risk (HR = 3.4, *p* = 0.02).
Ghahremani et al. (2023a)	MBI-C	Functional connectivity (FC)	MBI + individuals showed diminished FC between PCC and MPFC (*p* = 0.0037) and ACC and left anterior insula (*p* = 0.028).
Gosselin et al. (2023)	NPI-Q	Hearing loss (HL)	Untreated HL linked to global MBI (ORadj. = 1.66, *p* < 0.001); treated HL associated with incident MBI (HRadj. = 1.29, *p* = 0.04).
Ghahremani et al. (2023b)	NPI	Plasma p-tau181 levels	MBI linked to increased plasma p-tau181 levels (*p* = 0.02); predicts cognitive decline and greater dementia incidence (*p* < 0.001).
Mudalige et al. (2023)	NPI	Sleep disorders (SD)	MBI bidirectionally related to SD (HR = 3.04, *p* < 0.001); higher rate of developing MBI in those with SD (HR = 1.52, *p* < 0.001).
Ismail et al. (2023)	NPI, NPI-Q	Aβ42 levels, p-tau, t-tau	MBI linked to reduced Aβ42 levels (*p* = 0.039) and elevated p-tau (*p* = 0.001); greater incidence of dementia (*p* < 0.001).
Tsai et al. (2023)	MBI-C (Taiwanese version)	Health-related quality of life (HR-QoL)	Lower HR-QoL correlated with higher MBI-C scores (*p* < 0.001) and subdomains of decreased motivation (*p* < 0.001).
Monchi et al. (2024)	MBI-C	Microstructure of connections	Disruptions in connections between left amygdala and putamen in PD-MBI patients; increased tissue radial diffusivity (*p* = 0.004).
Leow et al. (2024)	MBI-C	Depression, fasting glucose levels	MCI participants had higher MBI scores (*p* < 0.001); fasting glucose levels correlated with MBI-social domain (*p* < 0.001).
Matsuoka et al. (2024)	MBI-C	Loneliness	Higher burden of MBI linked to loneliness (*p* < 0.001); LS score strong indicator for MBI-C total score.
Matuskova et al. (2024)	MBI-C	APOE e4, BDNF Met genetic polymorphisms	Neither APOE e4 nor BDNF Met significantly impacted MBI severity; interaction did not influence MBI scores.
Richey et al. (2024)	NPI-Q	Head trauma history	Greater frequency of MBI symptoms linked to head trauma, particularly in affective dysregulation (OR = 1.83) and impulse dyscontrol (OR = 1.74).

AD, Alzheimer’s disease; ADNI, Alzheimer’s Disease Neuroimaging Initiative; aMCI, Amnestic mild cognitive impairment; ACC, anterior cingulate cortex; APOE, apolipoprotein E; AxD, axial diffusivity; CI, confidence interval; CN, cognitively normal; DMN, default mode network; FA, fractional anisotropy; FC, functional connectivity; FI, Frailty Index; GDS, Geriatric Depression Scale; GMV, Grey matter volume; HC, healthy controls; HHIE-S, Hearing Handicap for the Elderly–Screening; HIS, Hachinski Ischemic Score; HL, hearing loss; H&Y, Hoehn and Yahr; HV, Hippocampus volume; LC, locus coeruleus; LMEs, Longitudinal mixed-effects models; LOC, Lost of Consciousness; LS, loneliness scale; MD, mean diffusivity; MCI, mild cognitive impairment; MBI-C, Mild Behavioral Impairment Checklist; MPFC, medial prefrontal cortex; NPS, neuropsychiatric symptoms; ORadj., adjusted odds ratio; PCC, posterior cingulate cortex; PD-MBI, Parkinson’s disease-mild behavioral impairment; PFC, prefrontal cortex; RD, radial diffusivity; ROI(s), region(s) of interest; SAN, salience network; SAS, Self-rating Anxiety Scale; SD, sleep disturbance; SN, salience network; SUVR, Standard Uptake Value Ratio; TBI, traumatic brain injury; UPDRS, Unified Parkinson’s Disease Rating Scale; VBM, voxel-based morphometry; WMH, white matter hyperintensities.

### 3.4 Demographic factors

A total of eight studies examined demographic factors associated with MBI. Gender was investigated in four studies, with two reporting significant associations: Wolfova et al. (2022) and Mortby et al. (2018), who found a higher prevalence of MBI in males, particularly for decreased motivation (*p* = 0.049) and impulse dyscontrol (*p* < 0.001). Conversely, studies from Yoo et al. (2020) in PD patients and Rao et al. (2020a) in an Indian population, found no gender differences.

Educational attainment was analyzed in three studies, with two reporting shorter education years in MBI cases (Shu et al., 2021b; Lang et al., 2020), while one found no association (Yoo et al., 2020). Marital status was evaluated in two studies, with one identifying it as a protective factor (Rao et al., 2020a). Age was a significant covariable in one out of three studies examining age effects, such as Miao et al. (2022) in cerebrospinal fluid biomarker analysis (*p* = 0.003).

### 3.5 Genetic factors

Five studies investigated genetic factors related to MBI. Andrews et al. (2018) found that the APOE ε4 allele was associated with affective dysregulation (OR = 1.65, *p* < 0.01), MS4A4A/MS4A6A polymorphisms with social inappropriateness, and BIN1/EPHA1 variations with abnormal perception or thought control disorders.

Ramezani et al. (2021) reported that the BDNF Met allele was associated with a higher likelihood of MBI in a Canadian population (*OR*: 4.38, *p* = 0.002). Creese et al. (2023) and Matuskova et al. (2024) both confirmed the link between the APOE ε4 allele and MBI risk. Stella et al. (2022) observed a non-significant positive correlation between APOE ε4 and MBI - C scores in non - demented older adults.

### 3.6 Cerebrospinal fluid and plasma markers

Five studies explored the association between MBI and amyloid beta (Aβ) proteins plasma levels. Miao et al. (2022) observed a link between a greater MBI total score and a lower plasma Aβ42/Aβ40 ratio, suggesting a potential role for Aβ imbalance in MBI development. Furthermore, elevated levels of P-tau181, another biomarker associated with Alzheimer’s disease pathology, were linked to MBI (Johansson et al., 2021; Ghahremani et al., 2023b). Recent research further supported with similar findings (Ismail et al., 2023).

Beyond Alzheimer’s-specific markers, Rao et al. (2020b) identified vitamin D deficiency and high triglyceride levels as potential risk factors for MBI in an Indian population.

### 3.7 Neuroimaging and brain morphology

A total of 16 studies explored neuroimaging correlates of MBI. Brain atrophy and gray matter volume were examined in 6 studies, all reporting significant associations. Yoon et al. (2019) linked reduced right middle temporal cortex volume to higher MBI-C scores (*p* < 0.01), while Shu et al. (2021b) and Matuskova et al. (2021) observed global gray matter and medial temporal lobe atrophy in MBI, respectively. Gill et al. (2021) found that MBI impulse dyscontrol correlated with Alzheimer’s-like atrophy patterns (*p* < 0.001), while Johansson et al. (2021) showed entorhinal cortex thickness strongly correlated with MBI-C scores (*p* < 0.001).

Functional connectivity alterations were reported in four studies. Lang et al. (2020) demonstrated reduced corticostriatal connectivity between the striatum and default mode/salience networks in Parkinson’s disease with MBI (*p* < 0.01), while Yoo et al. (2020) found decreased dopamine transporter availability in the anterior caudate and putamen (*OR*: 0.60–0.58, *p* < 0.01), directly associating anterior striatal pathology with memory dysfunction and motor deficits in MBI. In dementia-free individuals, Ghahremani et al. (2023a) and Lussier et al. (2020) reported diminished default mode network connectivity and increased frontal/parietal amyloid PET signal (*p* < 0.05), indicative of early neurodegenerative processes. Cassidy et al. (2022) found that locus coeruleus (LC) preservation may confer risk for MBI, especially in impulse control symptoms.

White matter and microstructural abnormalities were explored in three studies. Miao et al. (2021) and Yang et al. (2022) associated MBI with increased white matter hyperintensities (WMH, *p* = 0.01) and WMH-mediated gray matter atrophy, while Monchi et al. (2024) identified abnormal orbitofrontal-amygdala microstructure in MBI (*p* < 0.05).

Specific network dysfunction was reported in four studies, including frontoparietal control network deficits (Matsuoka et al., 2021; Shu et al., 2021a) and hippocampal memory system impairments (Yoon et al., 2021), both linked to MBI symptom severity.

### 3.8 Cognitive factors

Three studies examining the association between MBI and cognitive function consistently reported significant impairments. Yoon et al. (2019) demonstrated that the Parkinson’s disease patients with MBI group had significantly lower MoCA scores and z-scores across all five domains and the global score when compared to both healthy controls and Parkinson’s disease patients without MBI (*p* < 0.01). This evidence aligns with findings from studies in MCI cohorts (Creese et al., 2023; Leow et al., 2024), which observed similar cognitive decrements but were conducted in individuals with mild cognitive impairment rather than Parkinson’s disease populations.

### 3.9 Health status and comorbidities

A total of 13 studies explored the associations between MBI and health status or comorbidities. Regarding chronic diseases and multimorbidity, all 3 relevant studies found significant associations. Soo et al. (2021) reported that individuals with MCI and diabetes mellitus (DM) had a higher occurrence and severity of MBI. Rao et al. (2020a) also found an association between cerebrovascular risk factors, such as hypertension and diabetes, and MBI. Stella et al. (2022) suggested that comorbidities, including urinary incontinence and multimorbidity, may have a connection with MBI.

For the relationship between neuromotor disorders and MBI, the two relevant studies both obtained significant results. Baschi et al. (2019) found that in newly diagnosed Parkinson’s disease patients, motor disability (measured by H&Y stage, *p* < 0.01) and antidepressant use (*p* < 0.01) were significantly associated with MBI. Yoo et al. (2020) further discovered that a decrease in dopamine transporter availability in the anterior striatum (*OR*: 0.58, *p* = 0.008) was related to MBI motor deficits in Parkinson’s disease patients.

In the field of sensory impairment and interventions, three studies (Gosselin et al., 2019; Gosselin et al., 2022; Gosselin et al., 2023) showed significant associations.

Gosselin et al. (2019) explored the relationship between hearing loss and neuropsychiatric symptoms (NPS, a core component of the MBI diagnostic framework). The study found that individuals with hearing impairment exhibited significantly more NPS in the MBI domains of apathy (*χ*^2^ = 7.62, *p* = 0.006) and impulse dyscontrol (*χ*^2^ = 4.41, *p* = 0.036) compared to those with normal hearing. Notably, this association extended to the overall MBI burden: hearing loss was linked to a more significant global score on the MBI-C, indicating that compromised auditory function may manifest as specific NPS within the MBI construct, rather than simply correlating with isolated psychiatric symptoms. Gosselin et al. (2022) later reported a relationship between apathy and affective dysregulation. Then Gosselin et al. (2023) noted that the use of hearing aids was associated with a reduced prevalence of MBI (*p* = 0.04).

Concerning trauma and quality of life, the three (Richey et al., 2024; Bray et al., 2022; Tsai et al., 2023) relevant studies both reached significant conclusions. Richey et al. (2024) linked prior head injury to a higher prevalence of symptoms in specific MBI domains, particularly affective dysregulation and impulse dyscontrol (*OR*: 1.74). Traumatic brain injury history also played a pivotal role (*p* = 0.024) (Bray et al., 2022). Higher scores on the MBI-C were associated with decreased HR-QoL (*p* < 0.001) (Tsai et al., 2023). Additionally, this review also noticed associations between MBI and frailty (Fan et al., 2020; Guan et al., 2022).

### 3.10 Psychosocial factors

Three studies (Matsuoka et al., 2024; Rao et al., 2020a; Mudalige et al., 2023) investigated the association between MBI and psychosocial factors, all reporting significant correlations. Matsuoka et al. (2024) reported that loneliness was a predictor of the overall MBI-C score (*p* < 0.001) and specific MBI domains including decreased motivation, affective dysregulation, and abnormal perception. Depression was also found to be significantly higher in individuals with MBI compared to those without MBI (*p* = 0.007) (Rao et al., 2020a). On the other hand, sleep disturbance (SD) showed a bidirectional relationship with MBI (*p* < 0.001) (Mudalige et al., 2023).

## 4 Discussion

This review systematically synthesizes 41 studies to characterize risk factors in MBI, focusing on demographic correlates, biomarkers, neuroimaging features, and comorbid associations. Key findings highlight MBI’s multifactorial mechanisms, closely linked to AD pathological markers (e.g., reduced Aβ42/Aβ40 ratio, elevated p-tau181) and neurodegenerative structural changes like cortical atrophy and functional connectivity disruptions. Demographic analyses reveal heterogeneous effects of age, education, and sex on MBI risk, with potential generalizability limitations due to selection bias between clinical (predominantly older, MCI/PD populations) and community (broader age, higher cognitively normal individuals) samples. CSF and plasma biomarkers suggest MBI may represent a prodromal stage of AD, while neuroimaging underscores its association with early brain network dysfunction. Despite methodological heterogeneities and sample biases, this synthesis strengthens MBI as a critical early intervention target. Future research should prioritize integrative biopsychosocial models and cross-population validation to advance mechanistic understanding and clinical translation.

### 4.1 Methodological limitations of included studies

The studies included in this review have several methodological limitations. First, the heterogeneity in study designs, ranging from cross-sectional to longitudinal approaches (Tsai et al., 2023; Ismail et al., 2023), complicates causal inference. Reliance on self-reported measures and variability in MBI assessment tools may introduce reporting bias and limit generalizability. Additionally, relatively small sample sizes in some studies (Gosselin et al., 2019; Matsuoka et al., 2021) restrict the statistical power to detect subtle associations.

Regarding study settings, clinical studies may introduce selection bias, as they enroll individuals with higher prevalence and severity of neuropsychiatric symptoms (NPS) compared to community samples (Hu et al., 2023), potentially skewing sample demographics. Specifically, clinical studies (28 studies) predominantly included individuals aged ≥65 years, with more participants having MCI and PD, whereas community-related and studies (13 studies) covered ages 44–100 years, with more cognitively normal participants (Table 1). This age stratification may amplify associations between advanced age and neurodegenerative markers—for example, the negative correlation between age and plasma Aβ42/Aβ40 ratio (Miao et al., 2022) was not found in community cohorts.

### 4.2 Assessment method variability

The nature of MBI assessment tools used across studies adds complexity to the findings. Different tools capture varying aspects of MBI, which can affect the demographic profile of identified cases. For example, the MBI-C, as used by Ismail et al. (2017a), emphasizes specific behavioral domains, such as mood and social engagement, potentially influencing the perceived demographic risk factors. In contrast, the NPI-Q was designed to assess a broader range of neuropsychiatric symptoms, necessitating the transformation of NPI-Q scores into MBI domains to estimate MBI occurrence (Cummings et al., 1994). These differing assessment approaches may lead to heterogeneous estimates of MBI prevalence and symptom severity (Hu et al., 2023). For instance, Cui et al. (2024) found that MBI-C demonstrated 100% sensitivity in detecting early behavioral changes in behavioral variant frontotemporal dementia (bvFTD), outperforming NPI-Q. Conversely, NPI-Q’s emphasis on symptom severity makes it more effective for identifying severe neuropsychiatric symptoms. This review synthesized key tool characteristics—including assessed domains, target populations, and scoring logic. Based on the reviewed literature, MBI - C is often utilized in contexts focusing on early behavioral changes, whereas NPI - Q is more commonly associated with severe symptom assessment, reflecting their distinct design intents and application scenarios.

### 4.3 Demographic factors

The demographic characteristics of study populations in our systematic review present a diverse picture, with studies categorized by setting (clinical, community, and both) and age ranges (from adult/mid-life to older adults 60 and above). This diversity is crucial for understanding the varying risk profiles for MBI.

#### 4.3.1 Age and education

Age is a critical factor in understanding MBI, as cognitive decline and dementia risk typically increase with advancing age. Studies have shown that older adults, particularly those over 60, are at a heightened risk for MBI (Rao et al., 2020a). Furthermore, educational attainment plays a significant role; lower educational levels have been associated with a higher prevalence of MBI (Ye et al., 2023). This relationship suggests that educational interventions could be beneficial in mitigating MBI risk, particularly in populations with lower educational backgrounds.

#### 4.3.2 Gender differences

The review also highlights evidence of higher MBI rates in males (Mortby et al., 2018; Wolfova et al., 2022). However, findings are inconsistent, Yoo et al. (2020) in Parkinson’s disease and Rao et al. (2020a) in an Indian population observed no gender differences. This limited evidence base—comprising just 4 studies—underscores the need for caution when interpreting gender disparities in MBI. The discrepancy across literature reflects the complex role of gender in MBI, necessitating more nuanced investigations (e.g., integrating hormonal factors, sex-specific brain structural differences) to clarify underlying mechanisms.

While previous research has established a higher risk for Alzheimer’s disease in females (Xiong et al., 2022), our review indicates that MBI, as a potential precursor to dementia, may exhibit a different gender trend, with a noted prevalence in males. This observation prompts a critical examination of the biological and psychological factors that may contribute to such disparities. Additionally, these findings may be influenced by cultural and social factors, which can shape the expression and recognition of MBI across different populations. Skup et al. (2011) propose that differences in brain structure and function between genders could lead to varied patterns of brain atrophy, potentially influencing the expression of MBI. Additionally, societal norms and expectations may shape the manifestation of MBI in males, with externalizing behaviors such as aggression or impulsivity being more readily identified as early signs of MBI (Strüber et al., 2020). The contrasting findings from studies within our review, including those that report no significant gender differences (Rao et al., 2020a), highlight the need for a more comprehensive approach to studying gender influences on MBI.

### 4.4 Gene and protein

While the consistency of genetic associations across diverse populations presents a promising foundation for understanding MBI (Kim and Song, 2020; Deming et al., 2020), it also mandates a deeper exploration of the underlying biological underpinnings. For instance, the presence of the APOE ε4 allele is a well-established risk factor for cognitive decline in Alzheimer’s disease, and its consistent association with MBI across populations suggests a shared biological pathway that warrants further investigation. Additionally, the intricate interplay among genetics, lifestyle factors—including diet, physical activity, and cognitive engagement—and their combined influence on the risk and progression of MBI cannot be overlooked. Unraveling these complex mechanisms is essential for developing targeted interventions and personalized prevention strategies.

### 4.5 Cerebrospinal fluid and plasma markers

The association between MBI and cerebrospinal fluid biomarkers highlights their role in identifying early neurodegenerative processes, consistent with MBI’s proposed status as a prodromal stage. These findings collectively highlight the multifactorial nature of MBI, which shares similarities with mild cognitive impairment (MCI) (Li et al., 2016) and subjective cognitive decline (SCD) (Wolfsgruber et al., 2015). SCD and MCI are widely considered to progress to Alzheimer’s disease (AD), and the similar multifactorial nature of MBI implies that it may play a similar role in the neurodegenerative disease process. The decreased Aβ42/Aβ40 ratio in CSF (Miao et al., 2022) and increased p-tau181 levels (Ghahremani et al., 2023b) suggest that MBI may be associated with the amyloid cascade and tau phosphorylation in Alzheimer’s disease. An imbalance in the Aβ42/Aβ40 ratio leads to Aβ aggregation, forming amyloid plaques, which in turn cause neuronal toxicity, disrupt neuronal connections, and affect neurotransmission. This may be one of the reasons for the behavioral and cognitive changes in MBI patients. Elevated p-tau181 indicates excessive tau phosphorylation, leading to neurofibrillary tangles that disrupt neuronal function and induce cell death.

These pathological processes position MBI as an early window for neurodegeneration, supporting its role as a prodromal stage of diseases like AD. Monitoring CSF biomarkers such as Aβ42/Aβ40 and p-tau181 enables early identification of MBI, facilitating timely interventions that may include biomarker-guided screening, targeted therapies for amyloid/tau pathology, and lifestyle modifications to modulate neuroplasticity. Integrating these biomarkers into clinical practice can more effectively identify at-risk individuals, providing opportunities for precision interventions aimed at slowing disease progression.

### 4.6 Neuroimaging and brain morphology

Neuroimaging findings provide critical insights into the structural and functional brain alterations underlying MBI, linking behavioral symptoms to neurodegenerative pathways. This review encompasses 16 neuroimaging studies, with 14 of them thoroughly analyzed within the neuroimaging context (Matsuoka et al., 2023). The remaining two studies introduce alternative methodological perspectives that have yet to be fully integrated into our narrative (Monchi et al., 2024; Ghahremani et al., 2023a). These innovative contributions reinforce the established consensus that foundational pathological changes observed in dementia may serve as precursors to the development of MBI. This alignment between recent and existing research supports the notion that early stages of neurodegeneration are not only associated with but may also instigate the behavioral manifestations of MBI.

Furthermore, parallels drawn with neuroimaging findings in the context of subjective cognitive decline (SCD) (Wang et al., 2020) prompt consideration of a potential bidirectional relationship between neurobiological changes and MBI. Neuroimaging techniques, with their ability to visualize brain structure and function, can provide insights into how the cerebral substrates of dementia contribute to MBI. Conversely, the behavioral symptoms characteristic of MBI may reflect or even contribute to the neurobiological changes captured by neuroimaging. This perspective opens avenues for future research to explore the intricate dynamics between MBI and neuroimaging markers of early neurodegeneration, potentially leading to the development of novel diagnostic and therapeutic strategies.

### 4.7 Cognitive factors

Cognitive decline is increasingly recognized as a precursor to dementia, and the presence of MBI may serve as an early indicator of this process (Ismail et al., 2021). The current systematic review underscores the complex interplay between cognitive status and MBI. Previous studies have identified MBI as a potential marker of cognitive impairment, suggesting that these conditions are interconnected rather than isolated (Jin et al., 2023; Rouse et al., 2021). Moreover, research by Mudalige et al. (2023) demonstrates that the relationship between MBI and cognitive decline is not merely concurrent but also longitudinal, particularly in the context of PD. This longitudinal association implies that MBI may not only reflect the current cognitive state but also predict future cognitive trajectories, underscoring its utility as a prognostic tool.

### 4.8 Health status and comorbidities

The correlation between health status, comorbidities, and MBI is a critical lens through which to view the complex interplay of factors contributing to neurocognitive health. Research suggests that systematic health influences cognitive-behavioral manifestations. Furthermore, the connection between hearing loss and neuropsychiatric symptoms (Gosselin et al., 2019; Gosselin et al., 2023) indicates that sensory impairments may profoundly affect on cognitive-behavioral health, with interventions like hearing aids potentially mitigating MBI prevalence.

The link between past head trauma and MBI symptoms (Richey et al., 2024; Bray et al., 2022) emphasizes the importance of considering an individual’s medical history in the context of neurocognitive disorders. Additionally, associations between MBI and health-related quality of life (Tsai et al., 2023), as well as frailty (Guan et al., 2022; Fan et al., 2020), highlight the multifaceted nature of MBI risk factors.

### 4.9 Psychosocial factors

Psychosocial factors are integral to the complex interplay of influences on MBI. The interactions among these factors, as highlighted by Wakefield et al. (2020), suggest that they could collectively form a foundational psychological framework for future MBI research. A history of depression or current depressive symptoms has been identified as a risk factor for cognitive impairment (Gallagher et al., 2018), and evidence suggests that this relationship may extend to MBI (Rao et al., 2020a). While depression is epidemiologically linked to MBI, the core behavioral and cognitive changes in MBI may be driven by neurodegenerative mechanisms independent of depression: Rock et al. (2014) showed that individuals with a history of depression exhibit persistent cognitive impairments even after depressive symptoms resolve, with these impairments overlapping with MBI’s core symptoms (e.g., decreased motivation, social inappropriateness). This suggests MBI may represent a distinct pathological state from depression, though their trajectories may interact bidirectionally—depression potentially increasing MBI risk through inflammatory or neurotransmitter pathways, while MBI-related behavioral changes exacerbating depressive symptoms. This underscores the multifaceted nature of MBI and the need to integrate psychosocial factors (e.g., depression history, social support) into its assessment and management.

### 4.10 Recommendation for future studies

Building on the identified gaps in MBI research, future studies should prioritize investigations that directly address the demographic, biological, and methodological limitations highlighted in this review. Lifestyle factors, such as smoking and excessive alcohol consumption (Ye et al., 2023), as well as rural residence (Jia et al., 2020), have been extensively studied within the context of dementia. However, their relevance to MBI has not yet been established, representing a notable gap in current research.

#### 4.10.1 Demographic factors

In parallel with demographic and lifestyle investigations, future research should prioritize gender-specific analyses to address the critical gap identified in this review, while also exploring lifestyle factors. Although lifestyle factors have been extensively studied in the context of dementia, there remains a gap in research regarding their relevance to MBI. Currently, an increasing number of MBI studies are being conducted on non-white populations. For example, Rao et al. (2020b) conducted a study on Indian population, and Leow et al. (2024) carried out research among Singaporeans. However, compared with studies on white populations, MBI research on non - white populations still has significant room for expansion in terms of sample size and research depth. Thus, quantitative studies using validated questionnaires are crucial for investigating how lifestyle factors (e.g., dietary habits, social engagement) and gender interact with MBI pathophysiology across ethnic groups, facilitating the development of culturally tailored intervention strategies.

#### 4.10.2 Gene and Protein factors

Building on the review’s emphasis on APOE ε4 allele associations and genetic heterogeneity (Kim and Song, 2020; Deming et al., 2020), future research should prioritize mechanistic studies of gene-lifestyle interactions. Future research should elucidate the biological pathways through which genetic variants contribute to MBI, potentially leading to novel therapeutic strategies. Studies could examine the interaction between specific genetic markers and lifestyle factors, such as diet and exercise, to determine their combined influence on MBI risk. A critical examination of current findings is necessary to clarify the specific pathways influenced by genetic factors.

Integrating genetic findings into the broader context of MBI etiology is essential, as MBI likely results from interactions among genetic, environmental, and lifestyle elements. This perspective is vital for investigating gene–environment interactions (Dunn et al., 2019) and their neurological implications, as well as assessing the feasibility of gene-based therapeutics to decelerate or prevent cognitive decline (Rao et al., 2023). Identifying individuals at higher genetic risk for MBI could facilitate early intervention and personalized treatment approaches, but genetic testing and counseling must be approached cautiously to ensure evidence-based practices that respect individual autonomy.

#### 4.10.3 Cerebrospinal fluid and plasma markers

Given the review’s demonstration that MBI shares pathological similarities with MCI and SCD (Wolfsgruber et al., 2015), future research should focus on biomarker validation in diverse MBI subgroups.

Future research should validate biomarkers in diverse populations and explore their predictive value in the progression from MBI to more severe cognitive impairments. Multicenter studies could assess the reliability and validity of CSF and plasma biomarkers (e.g., Aβ42/Aβ40 ratio, p-tau181) across different demographic groups, particularly in non-white populations where data are scarce. This is critical because current evidence from Miao et al. (2022) and Ghahremani et al. (2023b) is primarily from clinical samples, and their generalizability to community-dwelling individuals remains unestablished. Integrating these biomarkers into clinical practice could facilitate personalized medicine approaches, allowing for early intervention and tailored treatment strategies for individuals at risk of MBI, especially those with comorbidities like diabetes (Rao et al., 2020a)0.*4.10.4 Health Status.*

Building on the review’s findings of associations between MBI and frailty, hearing loss, and traumatic brain injury (Fan et al., 2020; Gosselin et al., 2023; Richey et al., 2024), future research should investigate synergistic effects of comorbidities. Health status plays a vital role in prognostic capacity (Bond et al., 2006) and should be considered in future MBI research. A multidisciplinary approach is needed to assess and manage MBI, considering various contributing factors and their implications for cognitive-behavioral health and quality of life. Studies could evaluate the impact of comorbid conditions, such as diabetes or hypertension, on MBI progression, utilizing clinical assessments and patient-reported outcomes. Specifically, longitudinal studies could explore whether hearing aid use (Gosselin et al., 2023) modifies the relationship between sensory impairment and MBI severity, the feasibility of which is supported by the review’s evidence of reversible risk factors.

These findings underscore the necessity for a multidisciplinary approach to MBI assessment and management, considering a wide array of health factors and their cognitive-behavioral implications. The accessibility of health status as an evaluative tool, compared to more intricate biomarkers, highlights its utility in early MBI detection and intervention.

#### 4.10.4 Psychosocial factors

Building on the review’s identification of loneliness, depression, and sleep disturbance as MBI correlates (Matsuoka et al., 2024; Rao et al., 2020a), future research should test targeted psychosocial interventions. Integrating psychosocial interventions into MBI management is essential, as they address emotional and social determinants of cognitive-behavioral health. Future research should explore specific psychosocial interventions, such as cognitive-behavioral therapy or community support programs, and their effectiveness in reducing MBI symptoms. Early identification and intervention for loneliness, depression, and sleep disturbances could be critical in preventing and treating MBI. Early identification and intervention for loneliness, as a predictor of MBI severity (Matsuoka et al., 2024), could be critical in preventing and treating MBI, especially given the bidirectional relationship with sleep disturbance (Mudalige et al., 2023).

Longitudinal studies are crucial for understanding the progression from risk factors to MBI, and research investigating the efficacy of interventions targeting modifiable risk factors is warranted. Ultimately, identifying a biopsychosocial model of MBI may enhance our understanding of this condition (Wade and Halligan, 2017).

### 4.11 Limitations and strengths of this review

This review acknowledges several limitations. First, potential publication bias and variations in study quality must be considered. While the primary focus has been on studies investigating risk factors for MBI, some included studies also examined outcomes that MBI might predict. Although these predictive outcomes are not the main emphasis of this review, they could offer valuable insights for future research (Vellone et al., 2022; Kassam et al., 2023; Ferraro et al., 2023; Ruthirakuhan et al., 2022). Second, language barriers limited the inclusion of potentially relevant research published in other languages. Third, the inherent heterogeneity across diverse settings and participant populations posed challenges in drawing definitive conclusions and precluded meta-analysis.

Despite these limitations, the review offers notable strengths. It is among the first to systematically synthesize 41 studies across genetic, neuroimaging, and psychosocial domains, providing a comprehensive overview of MBI risk factors. The rigorous search strategy (PRISMA guideline, five major databases, and citation searching) and standardized data extraction process enhance its methodological rigor. By highlighting gaps in non-white population research and longitudinal designs, the review identifies critical directions for future studies.

## 5 Conclusion

This review reveals a complex interplay of risk factors associated with Mild Behavioral Impairment (MBI). Key findings highlight the significant contributions of genetic predispositions, gender differences, pre-existing cognitive decline, sensory impairments—particularly hearing loss—comorbid conditions such as diabetes, observable brain structural changes, and psychosocial factors including loneliness. The heterogeneity of these factors underscores the necessity for a multifaceted approach to the diagnosis, management, and prevention of MBI.

Future research should prioritize a more detailed investigation into the relative contributions of these identified risk factors, focusing on their interactions and cumulative effects on the development of MBI. This includes exploring potential mediating and moderating variables that may influence the trajectory from MBI to more severe cognitive decline. Such research is crucial for developing effective screening tools and personalized interventions aimed at preventing or delaying the progression to dementia.

## Data Availability

The original contributions presented in the study are included in the article/Supplementary material, further inquiries can be directed to the corresponding author/s.
