# Supplementary material for: Risk factors of mild behavioral impairment: a systematic review
Source: Front Psychol. 2025 Jun 27;16:1586418. doi: 10.3389/fpsyg.2025.1586418 (PMC12247176; doi:10.3389/fpsyg.2025.1586418)

**Supplementary Table** **S1.** Comprehensive search strategy employed in each database.

| Source | Search Strategy | Hits Retrieved |
| --- | --- | --- |
| PubMed | ("Cognitive Dysfunction"[MeSH Terms] OR ("Cognitive Dysfunction"[MeSH Terms] OR ("cognitive"[All Fields] AND "dysfunction"[All Fields]) OR "Cognitive Dysfunction"[All Fields] OR ("cognitive"[All Fields] AND "impairments"[All Fields]) OR "cognitive impairments"[All Fields] OR ("Cognitive Dysfunction"[MeSH Terms] OR ("cognitive"[All Fields] AND "dysfunction"[All Fields]) OR "Cognitive Dysfunction"[All Fields] OR ("cognitive"[All Fields] AND "disorder"[All Fields]) OR "cognitive disorder"[All Fields]) OR ("Cognitive Dysfunction"[MeSH Terms] OR ("cognitive"[All Fields] AND "dysfunction"[All Fields]) OR "Cognitive Dysfunction"[All Fields] OR ("mild"[All Fields] AND "cognitive"[All Fields] AND "impairment"[All Fields]) OR "mild cognitive impairment"[All Fields]) OR ("Cognitive Dysfunction"[MeSH Terms] OR ("cognitive"[All Fields] AND "dysfunction"[All Fields]) OR "Cognitive Dysfunction"[All Fields] OR ("cognitive"[All Fields] AND "decline"[All Fields]) OR "cognitive decline"[All Fields]) OR ("Cognitive Dysfunction"[MeSH Terms] OR ("cognitive"[All Fields] AND "dysfunction"[All Fields]) OR "Cognitive Dysfunction"[All Fields] OR ("mental"[All Fields] AND "deterioration"[All Fields]) OR "mental deterioration"[All Fields]) OR ("neurocognitive disorders"[MeSH Terms] OR ("neurocognitive"[All Fields] AND "disorders"[All Fields]) OR "neurocognitive disorders"[All Fields]) OR (("neuropsychiatric"[All Fields] OR "neuropsychiatrically"[All Fields] OR "neuropsychiatrics"[All Fields]) AND ("diagnosis"[MeSH Subheading] OR "diagnosis"[All Fields] OR "symptoms"[All Fields] OR "diagnosis"[MeSH Terms] OR "symptom"[All Fields] OR "symptom s"[All Fields] OR "symptomes"[All Fields]))))  AND  ("mild behavioral impairment"[All Fields] OR "mild behavioural impairment"[All Fields] OR "mild behavior impairment"[All Fields]) | 161 |
| Scopus | ( ALL ( "Cognitive Dysfunction" OR cognitive AND impairments OR cognitive AND disorder OR mild AND cognitive AND impairment OR cognitive AND decline OR mental AND deterioration OR neurocognitive AND disorders OR neuropsychiatric AND symptoms )  AND  ALL ( "mild behavioral impairment" OR "mild behavioural impairment" OR "mild behavior impairment" ) ) | 324 |
| Web of Science | "Cognitive Dysfunction" OR Cognitive Impairments OR Cognitive Disorder OR Mild Cognitive Impairment OR Cognitive Decline OR Mental Deterioration OR Neurocognitive Disorders OR Neuropsychiatric symptoms (All Fields) and "mild behavioral impairment" OR "mild behavioural impairment" OR "mild behavior impairment" (All Fields) | 185 |
| ScienceDirect | （"Cognitive Dysfunction" OR Cognitive Impairments OR Cognitive Disorder OR Mild Cognitive Impairment OR Cognitive Decline OR Mental Deterioration OR Neurocognitive Disorders OR Neuropsychiatric symptoms）AND ( "mild behavioral impairment" "mild behavioural impairment" "mild behavior impairment") | 210 |
| Embase | ((((((('cognitive dysfunction' OR cognitive) AND impairments OR cognitive) AND disorder OR mild) AND cognitive AND impairment OR cognitive) AND decline OR mental) AND deterioration OR neurocognitive) AND disorders OR neuropsychiatric) AND symptoms AND ('mild behavioral impairment' OR 'mild behavioural impairment' OR 'mild behavior impairment') | 169 |
|  |  | 1049 |
|  |  |  |

After importing into Endnote 20 library, with duplicated remove function.


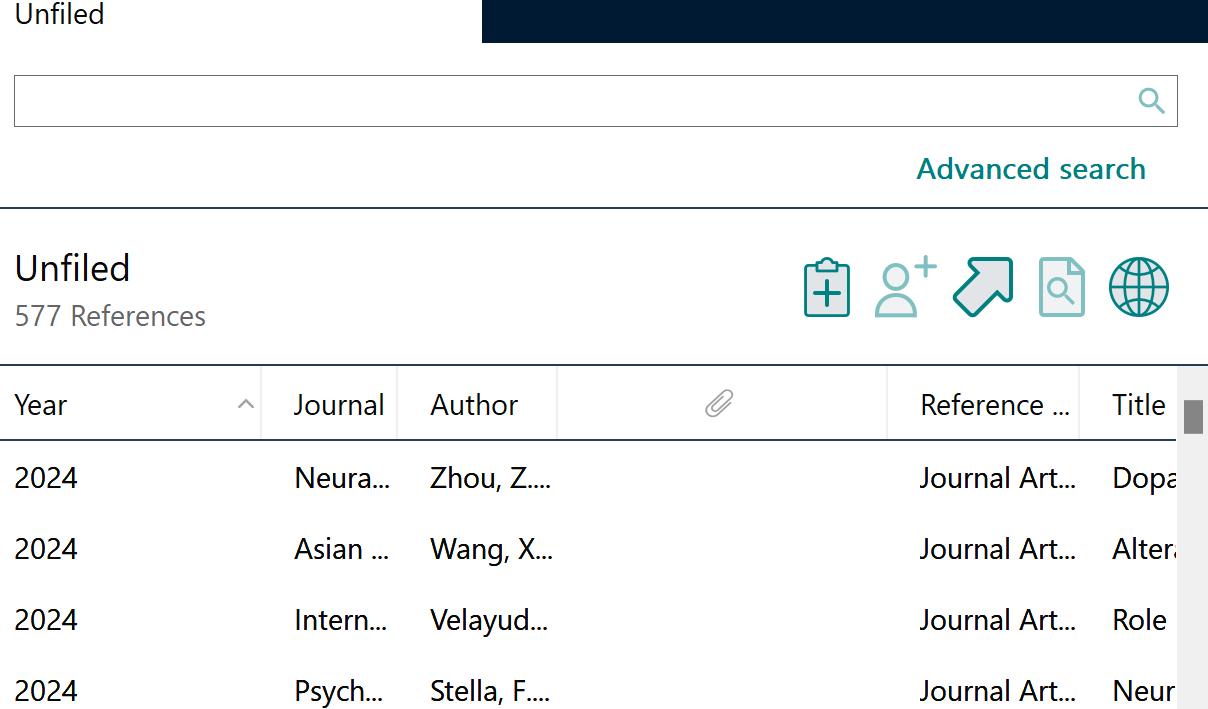


There are 577 references remained.

Date：2^nd^,May,2024
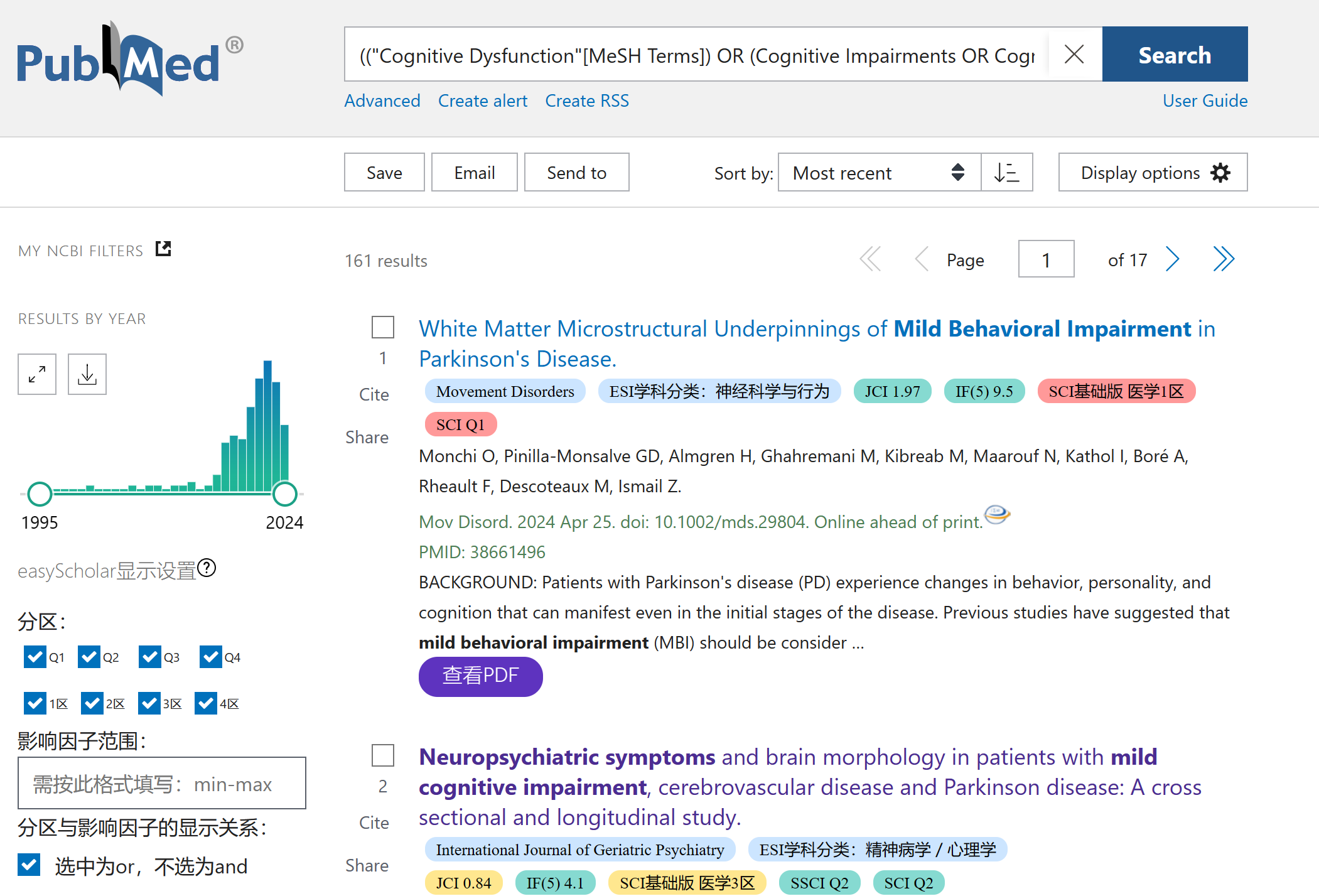


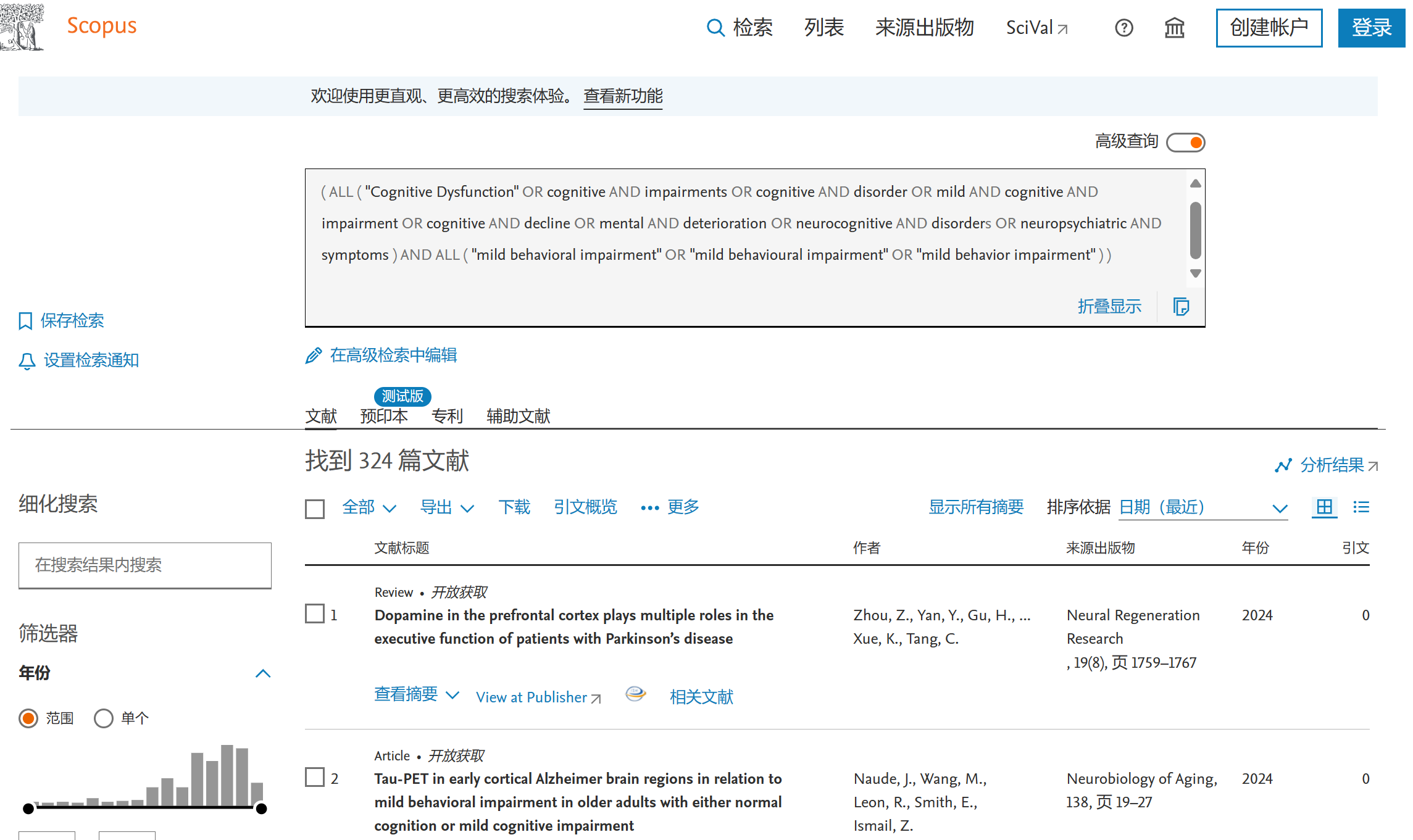


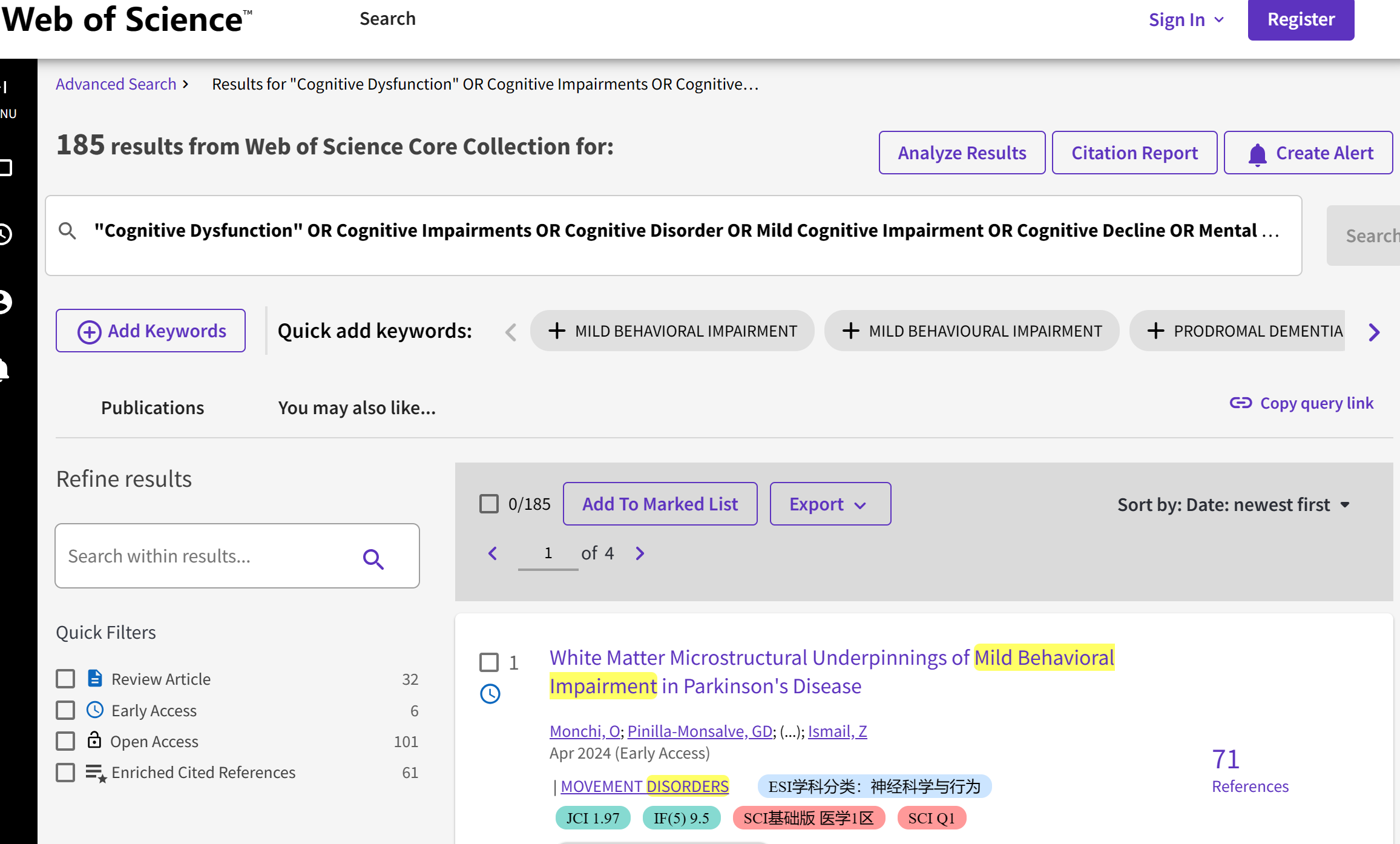


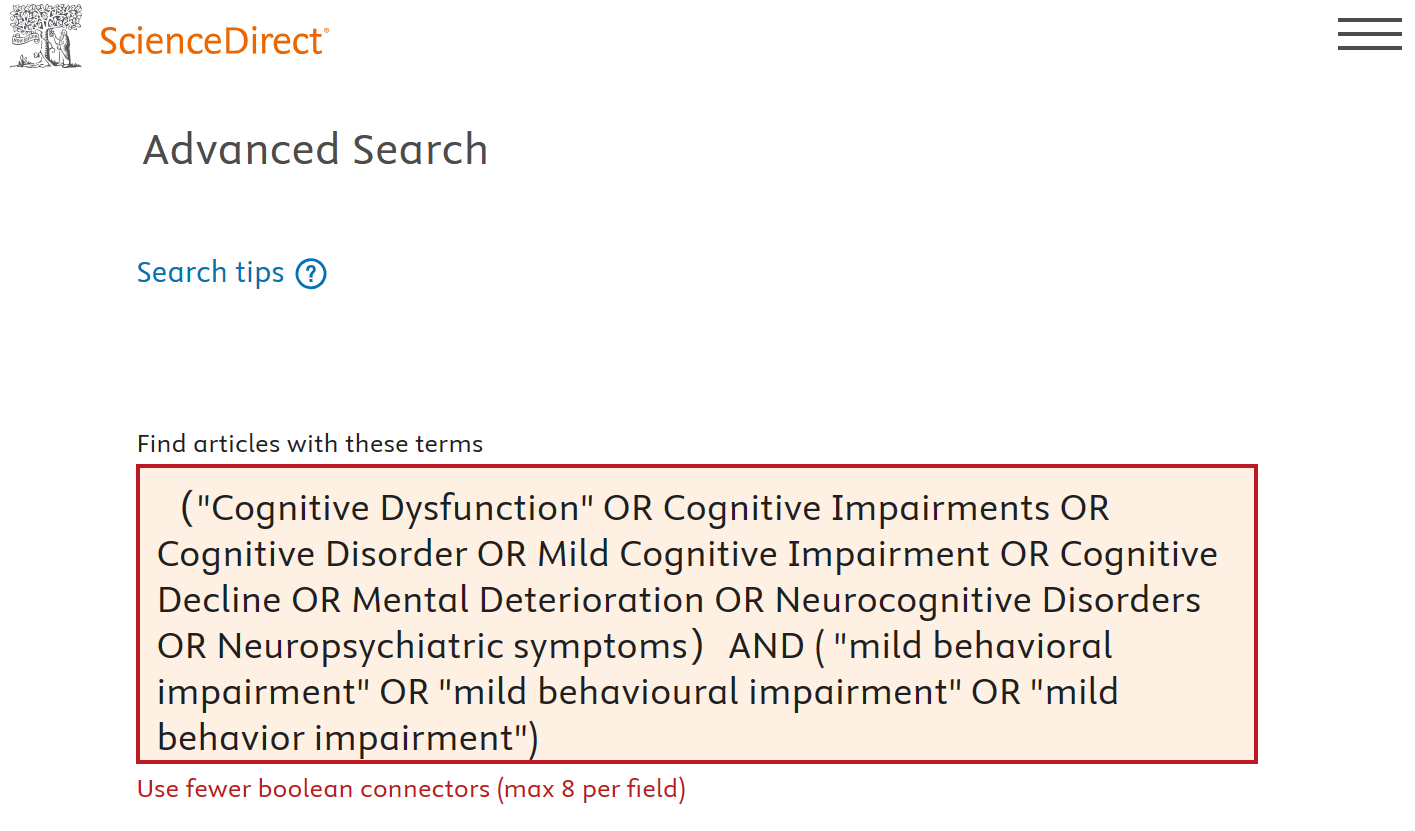


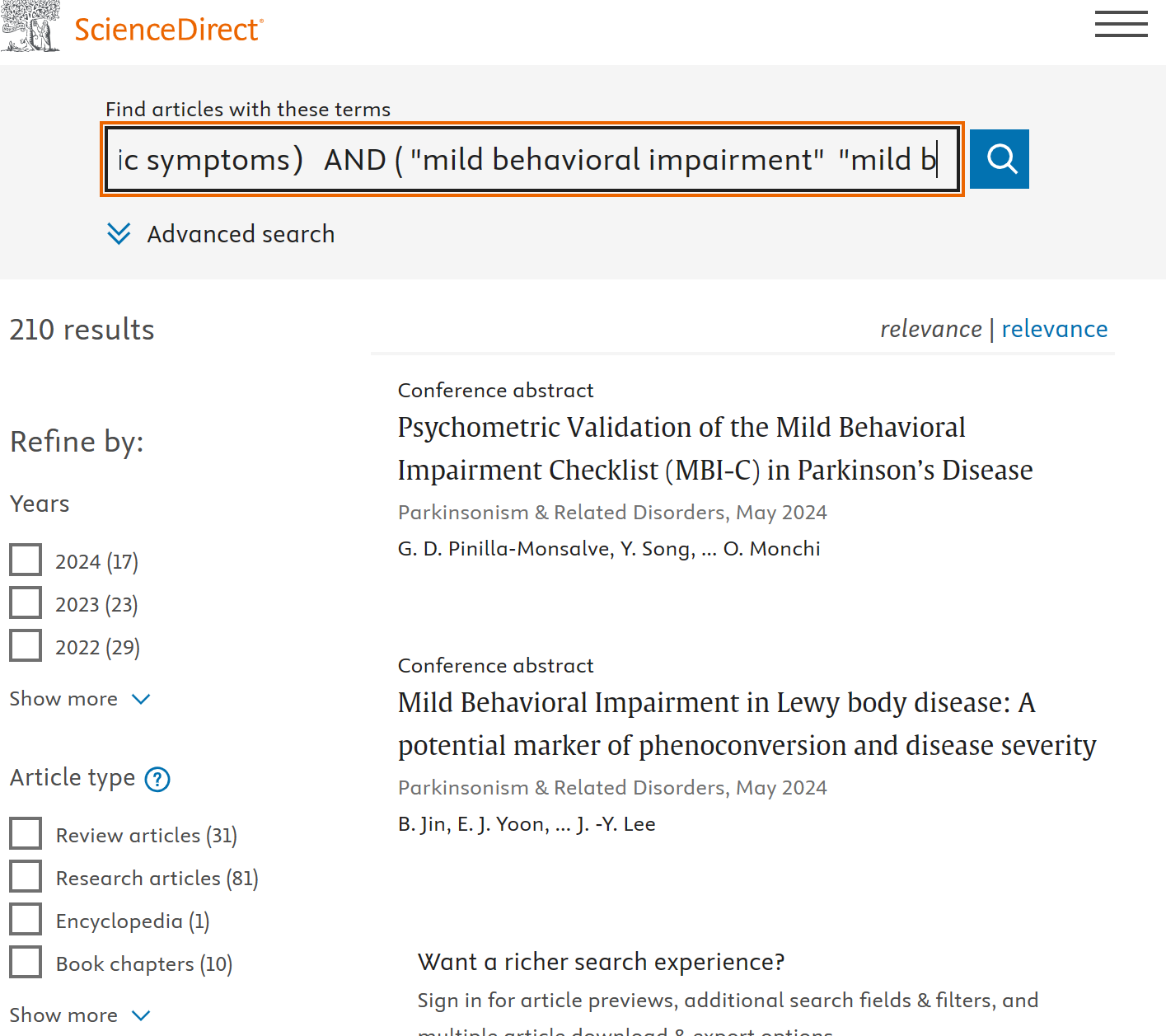


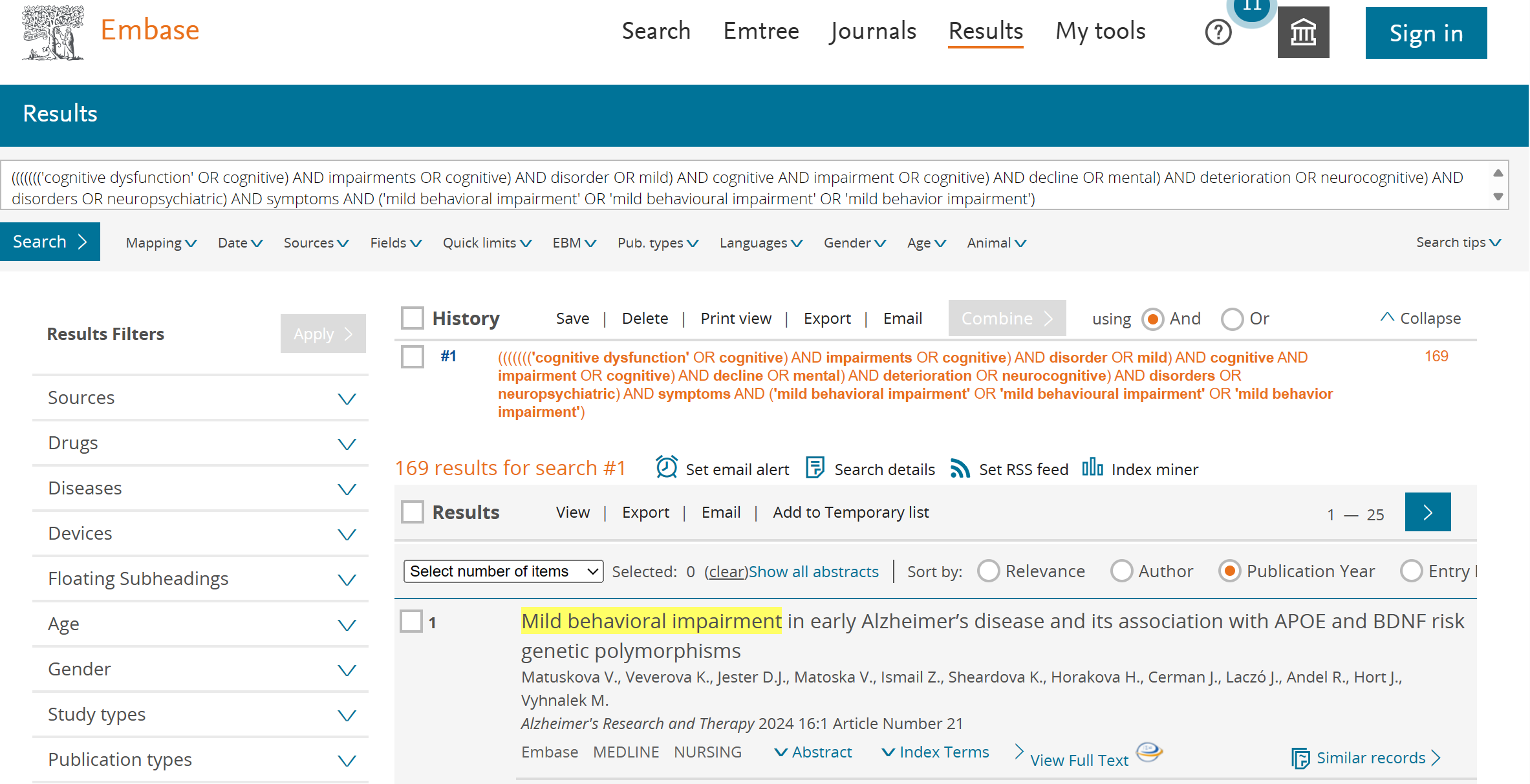

Supplement: Supplementary file 1 [file Supplementary_file_1.docx]
