# Supplementary material for: Risk factors of mild behavioral impairment: a systematic review
Source: Front Psychol. 2025 Jun 27;16:1586418. doi: 10.3389/fpsyg.2025.1586418 (PMC12247176; doi:10.3389/fpsyg.2025.1586418)
Supplement: Supplementary file 2 [file Supplementary_file_2.docx]

**Supplemental table S2: Quality Assessment of Cross-sectional Studies** (Joanna Briggs Institute Critical Appraisal Checklist for analytical cross sectional studies)

| Study | 1. Were the criteria for inclusion in the sample clearly defined? | 2. Were the study subjects and the setting described in detail? | 3. Was the exposure measured in a valid and reliable way? | 4. Were objective, standard criteria used for measurement of the condition? | 5. Were confounding factors identified? | 6. Were strategies to deal with confounding factors stated? | 7. Were the outcomes measured in a valid and reliable way? | 8. Was appropriate statistical analysis used? |
| --- | --- | --- | --- | --- | --- | --- | --- | --- |
| (Andrews et al. 2018) | Yes | Yes | Yes | Yes | Yes | Yes | Yes | Yes |
| (Mortby et al. 2018) | Yes | Yes | Yes | Yes | no | no | Yes | Yes |
| (Baschi et al. 2019) | Yes | Yes | Yes | Yes | Yes | Yes | Yes | Yes |
| (Yoon et al. 2019) | Yes | Yes | Yes | Yes | Yes | Yes | Yes | Yes |
| (Fan et al. 2020) | Yes | Yes | Yes | Yes | Yes | no | Yes | Yes |
| (Lang et al. 2020) | Yes | Yes | Yes | Yes | Yes | Yes | Yes | Yes |
| (Lussier et al. 2020) | Yes | Yes | Yes | Yes | Yes | Yes | Yes | Yes |
| (Rao, Chatterjee, et al. 2020) | Yes | Yes | Yes | Yes | Yes | Yes | Yes | Yes |
| (Rao, Thakral, et al. 2020) | Yes | Yes | Yes | Yes | Yes | no | Yes | Yes |
| (Yoo et al. 2020) | Yes | Yes | Yes | Yes | Yes | no | Yes | Yes |
| (Ramezani et al. 2021) | Yes | Yes | Yes | Yes | Yes | Yes | Yes | Yes |
| (Gill et al. 2021) | Yes | Yes | Yes | Yes | Yes | Yes | Yes | Yes |
| (Johansson et al. 2021) | Yes | Yes | Yes | Yes | Yes | Yes | Yes | Yes |
| (Matsuoka et al. 2021) | Yes | Yes | Yes | Yes | Yes | Yes | Yes | Yes |
| (Miao et al. 2021) | Yes | Yes | Yes | Yes | Yes | Yes | Yes | Yes |
| (Shu, Qiang, Yan, Ren, et al. 2021) | Yes | Yes | Yes | Yes | Yes | Yes | Yes | Yes |
| (Shu, Qiang, Yan, Wen, et al. 2021) | Yes | Yes | Yes | Yes | Yes | Yes | Yes | Yes |
| (Soo et al. 2021) | Yes | Yes | Yes | Yes | Yes | Yes | Yes | Yes |
| (Yoon et al. 2021) | Yes | Yes | Yes | Yes | Yes | Yes | Yes | Yes |
| (Bray et al. 2022) | Yes | Yes | Yes | Yes | Yes | Yes | Yes | Yes |
| (Cassidy et al. 2022) | Yes | Yes | Yes | Yes | Yes | Yes | Yes | Yes |
| (Matuskova et al. 2021) | Yes | Yes | Yes | Yes | Yes | Yes | Yes | Yes |
| (Gosselin et al. 2022) | Yes | Yes | Yes | Yes | Yes | Yes | Yes | Yes |
| (Miao et al. 2022) | Yes | Yes | Yes | Yes | Yes | Yes | Yes | Yes |
| (Stella et al. 2022) | Yes | Yes | Yes | Yes | Yes | no | Yes | Yes |
| (Guan et al. 2022) | Yes | Yes | Yes | Yes | Yes | Yes | Yes | Yes |
| (Yang et al. 2022) | Yes | Yes | Yes | Yes | Yes | Yes | Yes | Yes |
| (Ghahremani, Nathan, et al. 2023) | Yes | Yes | Yes | Yes | Yes | Yes | Yes | Yes |
| (Tsai et al. 2023) | Yes | Yes | Yes | Yes | Yes | Yes | Yes | Yes |
| (Monchi et al. 2024) | Yes | Yes | Yes | Yes | Yes | Yes | Yes | Yes |
| (Leow et al. 2024) | Yes | Yes | Yes | Yes | Yes | Yes | Yes | Yes |
| (Matsuoka et al. 2024) | Yes | Yes | Yes | Yes | Yes | Yes | Yes | Yes |
| (Matuskova et al. 2024) | Yes | Yes | Yes | Yes | Yes | Yes | Yes | Yes |

Website: Aromataris E, Munn Z (Editors). JBI Manual for Evidence Synthesis. JBI, 2020. Available from <https://synthesismanual.jbi.global>

**Supplemental S3: Quality Assessment of Cohort Studies (Joanna Briggs Institute Critical Appraisal Checklist for cohort studies)**

| Study | JBI-C1 | JBI-C2 | JBI-C3 | JBI-C4 | JBI-C5 | JBI-C6 | JBI-C7 | JBI-C8 | JBI-C9 | JBI-C10 | JBI-C11 |
| --- | --- | --- | --- | --- | --- | --- | --- | --- | --- | --- | --- |
| Gosselin et al. (2019) | Yes | Yes | Yes | Yes | Yes | Yes | Yes | no | no | no | Yes |
| Wolfova et al. (2022) | Yes | Yes | Yes | Yes | Yes | Yes | Yes | Yes | no | no | Yes |
| Creese et al. (2023) | Yes | Yes | Yes | Yes | Yes | Yes | Yes | Yes | no | no | Yes |
| Gosselin et al. (2023) | Yes | Yes | Yes | Yes | Yes | Yes | Yes | Yes | Yes | Yes | Yes |
| Ghahremani, Wang, et al. (2023) | Yes | Yes | Yes | Yes | Yes | Yes | Yes | Yes | Yes | no | Yes |
| Mudalige et al. (2023) | Yes | Yes | Yes | Yes | Yes | Yes | Yes | Yes | Yes | Yes | Yes |
| Ismail et al. (2023) | Yes | Yes | Yes | Yes | Yes | Yes | Yes | Yes | Yes | no | Yes |
| Richey et al. (2024) | Yes | Yes | Yes | Yes | Yes | Yes | Yes | Yes | no | no | Yes |

**Website:** Aromataris E, Munn Z (Editors). JBI Manual for Evidence Synthesis. JBI, 2020. Available from https://synthesismanual.jbi.global

JBI-C 1. Were the two groups similar and recruited from the same population? □ □ □ □

JBI-C 2. Were the exposures measured similarly to assign people to both exposed and unexposed groups? □ □ □ □

JBI-C 3. Was the exposure measured in a valid and reliable way? □ □ □ □

JBI-C 4. Were confounding factors identified? □ □ □ □

JBI-C 5. Were strategies to deal with confounding factors stated? □ □ □ □

JBI-C 6. Were the groups/participants free of the outcome at the start of the study (or at the moment of exposure)? □ □ □ □

JBI-C 7. Were the outcomes measured in a valid and reliable way? □ □ □ □

JBI-C 8. Was the follow up time reported and sufficient to be long enough for outcomes to occur? □ □ □ □

JBI-C 9. Was follow up complete, and if not, were the reasons to loss to follow up described and explored? □ □ □ □

JBI-C 10. Were strategies to address incomplete follow up utilized? □ □ □ □

JBI-C 11. Was appropriate statistical analysis used? □ □ □ □
